# Editorial: The role of neutrophils and its NETosis in autoimmunity and autoinflammation

**DOI:** 10.3389/fimmu.2022.1035624

**Published:** 2022-10-20

**Authors:** François Niyonsaba

**Affiliations:** ^1^Atopy (Allergy) Research Center, Juntendo University Graduate School of Medicine, Tokyo, Japan; ^2^Faculty of International Liberal Arts, Juntendo University, Tokyo, Japan

**Keywords:** LL-37, defensin, neutrophil, NET, autoimmunity, autoinflammation

Neutrophils, the most abundant innate immune sentinels, are equipped with a plethora of antimicrobial molecules and are the first cells to migrate toward sites of inflammation to eradicate a wide array of microbial pathogens and clear necrotic cellular debris. The antimicrobial factors used by neutrophils to kill pathogens include antimicrobial peptides (e.g., lactoferrin, human α-defensins, also known as human neutrophil peptides (HNPs), and cathelicidin LL-37), proteolytic enzymes, and reactive oxygen species (ROS) (1, 2). Neutrophils kill and prevent the dissemination of invading pathogens *via* phagocytosis; the release of toxic proteins from azurophilic (or primary) granules, specific (or secondary) granules, and gelatinase (or tertiary) granules; and the ensnarement of pathogens through the release of DNA in the form of neutrophil extracellular traps (NETs) (3).

NETs are web-like structures composed of decondensed chromatin associated with antimicrobial agents, such as antimicrobial peptides and myeloperoxidase (MPO) (1–3). Following activation, neutrophil chromatin decondensates and forms complexes with granular proteins, which are finally extruded into the extracellular space. Although NETs were originally found in neutrophils and thought to function as a trap to kill invading microorganisms, a number of recent reports have demonstrated that eosinophils, mast cells, macrophages, lymphocytes (T lymphocytes, B lymphocytes, and natural killer cells) and monocytes also possess structures that are similar to neutrophil NETs (4, 5). The formation of NETs is triggered by various stimuli, such as bacteria, viruses, fungi, protozoa, components of the bacterial cell wall (lipopolysaccharides), cytokines, calcium ionophores, and phorbol-12-myristate-13-acetate (PMA) (1–5).

The protective role of NETs in the host immune system was evidenced by the observation that neutrophils from patients with chronic granulomatous disease had a mutant NADPH oxidase and could not form ROS, leading to recurrent infections (6). Those neutrophils were unable to form NETs. Although the initial function of NETs is to protect against microbial infections, recent studies suggest that NETs may function as double-edged swords, as they not only serve as an antimicrobial defense and exhibit anti-inflammatory effects but also play important proinflammatory roles by promoting tissue damage, autoinflammation, and autoimmunity. Regarding the anti-inflammatory effects of NETs, these structures reduce the lipopolysaccharide-induced secretion of proinflammatory cytokines in macrophages and dendritic cells (DCs) (7, 8) and the expression of antigen-presenting molecules by DCs (8). NETs also induce the expression of CD69 and CD25 in T cells, resulting in the priming of CD4^+^ T cells (9) and enhancing the surface expression of neutrophil activation markers, such as CD62L and CD11b (1). In neutrophils, NETs also facilitate exocytosis of specific granules, azurophilic granules, and secretory vesicles and induce the generation of NADPH oxidase 2-derived ROS. Furthermore, neutrophils exposed to NETs exhibit enhanced phagocytic activity (1). The fact that NETs are able to activate various functions of neutrophils indicates the role of these structures in a self-amplification mechanism of modulation of inflammation.

Regarding the proinflammatory roles of NETs, the excessive NET release has been shown to contribute to various chronic inflammatory diseases and autoimmune diseases. Indeed, autoantibodies that recognize NET components, such as double-stranded DNA and citrullinated proteins, have been detected in autoimmune diseases, including vasculitis, psoriasis, systemic lupus erythematosus (SLE), and rheumatoid arthritis, indicating the involvement of NETs in these conditions (5, 10). Moreover, renal deposition of NETs has been observed in patients with glomerulonephritis, which is a complication of SLE and vasculitis. NETs promote proinflammatory activities by interacting with several immune cells. In addition to autoimmune diseases, NETs also contribute to the pathogenesis of acute and chronic inflammatory diseases. In patients with acute respiratory distress syndrome, the level of NETs in the blood has been shown to correlate with the severity of the disease and mortality (11). Remarkably, the inhibition of NETs resulted in reduced acute lung injury induced by bacteria and improved survival (12). In chronic inflammatory diseases, such as cystic fibrosis and chronic obstructive pulmonary disease, sustained infiltration of neutrophils and persistent NET formation have been observed and were associated with inflammation and disease severity (13). NETs contribute to the pathogenesis of inflammatory and autoimmune diseases because they can directly activate various immune cells. Indeed, NETs induce the production of type I interferon (IFN) by plasmacytoid DCs (pDCs) (14), cause the release of interleukin (IL)-1β and IL-18 from macrophages, and facilitate the secretion of other proinflammatory cytokines, including IL-6, IL-8, tumor necrosis factor-α, and B cell-activating factor, from macrophages and neutrophils (7, 15).

In addition to their direct effects, NETs may indirectly exhibit both beneficial and harmful activities on immune cells. Upon activation, neutrophils release antimicrobial peptides, such as cathelicidin LL-37, which is predominantly stored in specific (secondary) granules, and α-defensin-1-4 (HNP-1-4), which are present in azurophilic (primary) granules (16). Of note, LL-37 and HNPs bind to the surface of NETs. LL-37 and HNPs not only exhibit antimicrobial activities but also control infection by modulating various immune functions, such as the regulation of apoptosis, cell migration, phagocytosis, cytokine induction, and the production of ROS (17, 18). Furthermore, these peptides play a crucial protective role in lipopolysaccharide-induced peritonitis (19). However, because both LL-37 and HNPs chemoattract immune cells, such as monocytes, immature DCs, naïve CD4^+^ T cells, macrophages, and mast cells, they may also exacerbate inflammation (17, 20, 21). LL-37 complexes with self-DNA to activate pDCs and monocytes through Toll-like receptor (TLR)-9, while it complexes with self-RNA to activate pDCs through TLR-7 and myeloid DCs (mDCs) through TLR-8 (22). In neutrophils, the RNA : LL-37 complex, but not the DNA : LL-37 complex, promotes inflammation by enhancing the formation of NETs and releasing proinflammatory cytokines, such as IL-1β, IL-6, IL-8, and tumor necrosis factor-α (22). Remarkably, skin biopsies from patients with SLE show elevated levels of LL-37 in correlation with increased levels of IFN-α and pDCs (23). Neutrophils isolated from patients with SLE release self-DNA and LL-37 to form NETs, and NET-associated LL-37 complexes lead to sustained inflammation in SLE (24). Of note, autoantibodies to LL-37 have been shown to induce NET formation. Similarly, enhanced levels of RNA : LL-37 complexes were found in NETs and psoriatic skin and promoted cytokine production and NET release by neutrophils (22, 25). Interestingly, NETs derived from neutrophils that were stimulated with RNA–LL-37 complexes induced the *de novo* formation of NETs, leading to further inflammation (25). Although the complex of HNPs and DNA has been found to be insufficient to cause the production of IFN-α by pDCs, HNPs promote pDC activation by protecting against DNA degradation (25). Furthermore, anti-HNP autoantibodies in SLE patient sera trigger immunogenic self-DNA–HNP complexes. Additionally, neutrophils isolated from patients with SLE release more NETs than neutrophils obtained from healthy controls in response to anti-LL-37 and anti-HNP antibodies (24).

In conclusion, in addition to preventing infections and resolving inflammation, NETs are drivers of inflammation. These structures are important regulators of inflammation and autoimmunity and may be potential targets for therapeutic use in inflammatory and autoimmune diseases.

## Author contributions

The author has made a substantial, direct, and intellectual contribution to the work and has approved it for publication.

## Acknowledgments

The author wishes to thank the members of the Atopy (Allergy) Research Center for their comments and Michiyo Matsumoto for her secretarial assistance.

## Conflict of interest

The author declares that the research was conducted in the absence of any commercial or financial relationships that could be construed as a potential conflict of interest.

## Publisher’s note

All claims expressed in this article are solely those of the authors and do not necessarily represent those of their affiliated organizations, or those of the publisher, the editors and the reviewers. Any product that may be evaluated in this article, or claim that may be made by its manufacturer, is not guaranteed or endorsed by the publisher.
